# Identification of Natural Compound Inhibitors for Multidrug Efflux Pumps of *Escherichia coli* and *Pseudomonas aeruginosa* Using *In Silico* High-Throughput Virtual Screening and *In Vitro* Validation

**DOI:** 10.1371/journal.pone.0101840

**Published:** 2014-07-15

**Authors:** Vasudevan Aparna, Kesavan Dineshkumar, Narasumani Mohanalakshmi, Devadasan Velmurugan, Waheeta Hopper

**Affiliations:** 1 Department of Bioinformatics, School of Bioengineering, Faculty of Engineering & Technology, SRM University, Kattankulathur, Tamilnadu, India; 2 Centre of Advanced Study in Crystallography and Biophysics, University of Madras, Guindy Campus, Chennai, Tamilnadu, India; University of Cambridge, United Kingdom

## Abstract

*Pseudomonas aeruginosa* and *Escherichia coli* are resistant to wide range of antibiotics rendering the treatment of infections very difficult. A main mechanism attributed to the resistance is the function of efflux pumps. MexAB-OprM and AcrAB-TolC are the tripartite efflux pump assemblies, responsible for multidrug resistance in *P. aeruginosa* and *E. coli* respectively. Substrates that are more susceptible for efflux are predicted to have a common pharmacophore feature map. In this study, a new criterion of excluding compounds with efflux substrate-like features was used, thereby refining the selection process and enriching the inhibitor identification process. An in-house database of phytochemicals was created and screened using high-throughput virtual screening against AcrB and MexB proteins and filtered by matching with the common pharmacophore models (AADHR, ADHNR, AAHNR, AADHN, AADNR, AAADN, AAADR, AAANR, AAAHN, AAADD and AAADH) generated using known efflux substrates. Phytochemical hits that matched with any one or more of the efflux substrate models were excluded from the study. Hits that do not have features similar to the efflux substrate models were docked using XP docking against the AcrB and MexB proteins. The best hits of the XP docking were validated by checkerboard synergy assay and ethidium bromide accumulation assay for their efflux inhibition potency. Lanatoside C and diadzein were filtered based on the synergistic potential and validated for their efflux inhibition potency using ethidium bromide accumulation study. These compounds exhibited the ability to increase the accumulation of ethidium bromide inside the bacterial cell as evidenced by these increase in fluorescence in the presence of the compounds. With this good correlation between *in silico* screening and positive efflux inhibitory activity *in vitro*, the two compounds, lanatoside C and diadzein could be promising efflux pump inhibitors and effective to use in combination therapy against drug resistant strains of *P. aeruginosa* and *E. coli*.

## Introduction

Multidrug resistant bacteria resist a broad range of antimicrobials thereby reducing the treatment options and hence increasing the mortality. There is an increase in incidence of infectious diseases in developing countries where the use of antibiotics is high. This problem of antimicrobial resistance is of great concern. The World Health Organization has urged on to “evaluate the strategies to overcome and control the spread of antimicrobial resistant micro­organisms” [1]. *Pseudomonas aeruginosa* is an opportunistic Gram-negative bacterium, resistant to multiple drugs, mainly due to low permeability of its cell membrane. This reduced permeability is owed to two reasons, efflux pumps and low porin protein expression [2]. The major mechanism of resistance in these organisms is the efflux pumps, which have their substrate specificity based on their polarity [3]. Multidrug resistance in *Escherichia coli* is also a major difficulty in the treatment of the infectious diseases caused by them, with efflux pumps as one of the mechanisms of resistance. The multidrug efflux pumps are membrane proteins that are involved in the pumping out of antibiotics and are classified into the resistant nodulation division (RND) family, the major facilitator super family (MFS), the staphylococcal multi-resistance (SMR) and the multidrug and toxic compound extrusion (MATE) family [4]. *P. aeruginosa* and *E. coli* have efflux pumps that belong to the RND family. AcrAB-TolC and MexAB-OprM are RND pumps that form a tripartite assembly in the bacterial membrane, contributing to the intrinsic and acquired antibiotic resistance in *E. coli* and *P. aeruginosa* respectively. They confer resistance to a large array of drugs which include quinolones, macrolides, tetracycline, chloramphenicol, novobiocin, and β-lactam [5]. Deletion of MexAB-OprM in wild-type strain of *P. aeruginosa* had made the strain hypersusceptibile to many drugs [4], thus giving the scope for the development of agents that could possibly block the activity of these pumps thereby making the organisms susceptible to the drugs.

It is reported that combating the resistance could be done by targeting the mechanism responsible for it, in this case by developing specific inhibitors against the efflux pumps [6]. Compounds that could interact with specific efflux pump proteins could restore the organism's susceptibility to drugs. This approach could counteract pathogens that harbour efflux pumps and compounds, the efflux pump inhibitors (EPIs) can be used as chemotherapeutics, along with the antibiotics. As efflux pumps provide both innate and higher-level resistance to antibiotics in bacteria, EPIs should ideally increase the activity of an antibiotic in multidrug-resistant cells [7] and this indicates the significance for developing small-molecule inhibitors against efflux pumps. The EPIs can increase effectively increase the intracellular concentration of the drug to the level essential for its activity and hence reduce the minimal inhibitory concentration required for the antibiotic to kill the resistant organisms. Phenylalanine arginyl β-naphthylamide (PAβN; MC-207110) was the first EPI identified for *Pseudomonas* strain harboring a MexAB-OprM pump; this peptidomimetic compound has a competitive mechanism of inhibition [8]. Carbonyl cyanide m-chlorophenylhydrazone (CCCP) is an energy-dependent EPI that de-energizes membranes unlike PAβN which is more substrate specific [9]. CCCP is not exactly termed as an EPI because it is involved with the proton motive force that is necessary for the working of RND type pumps thereby indirectly inhibiting the efflux mechanism [10]. However both these compounds are not applicable to clinical use due to their toxic properties.

Phytochemicals, natural compounds produced by plants have a very weak antimicrobial effect but still have the capacity to fight plant pathogens. This was due to a mechanism called synergy adopted by the plants [11]. These phytochemicals have minimal or almost no toxicity when used clinically and could be used in overcoming drug resistance in bacteria by blocking multidrug efflux pumps. A well known example is the plant alkaloid reserpine isolated from the roots of *Rauwolfia vomitoria* Afz which showed EPI activity against the Bmr efflux pump of *Bacillus subtilis* [12]. Porphyrin pheophorbide, silybin, methoxylated flavones and isoflavone are some of the phytochemicals that have demonstrated synergistic activity against NorA efflux harboring *Staphylococcus aureus* strains [13], [14]. All these compounds are EPIs of Gram-positive bacteria, particularly *S. aureus*, but for Gram-negative organisms like *P. aeruginosa*, *E. coli* and *Acinetobacter*, it is difficult to identify compounds with EPI activity because of their intrinsic resistance due to the presence of thick, lipophilic outer membrane [15].

**Figure 1:**
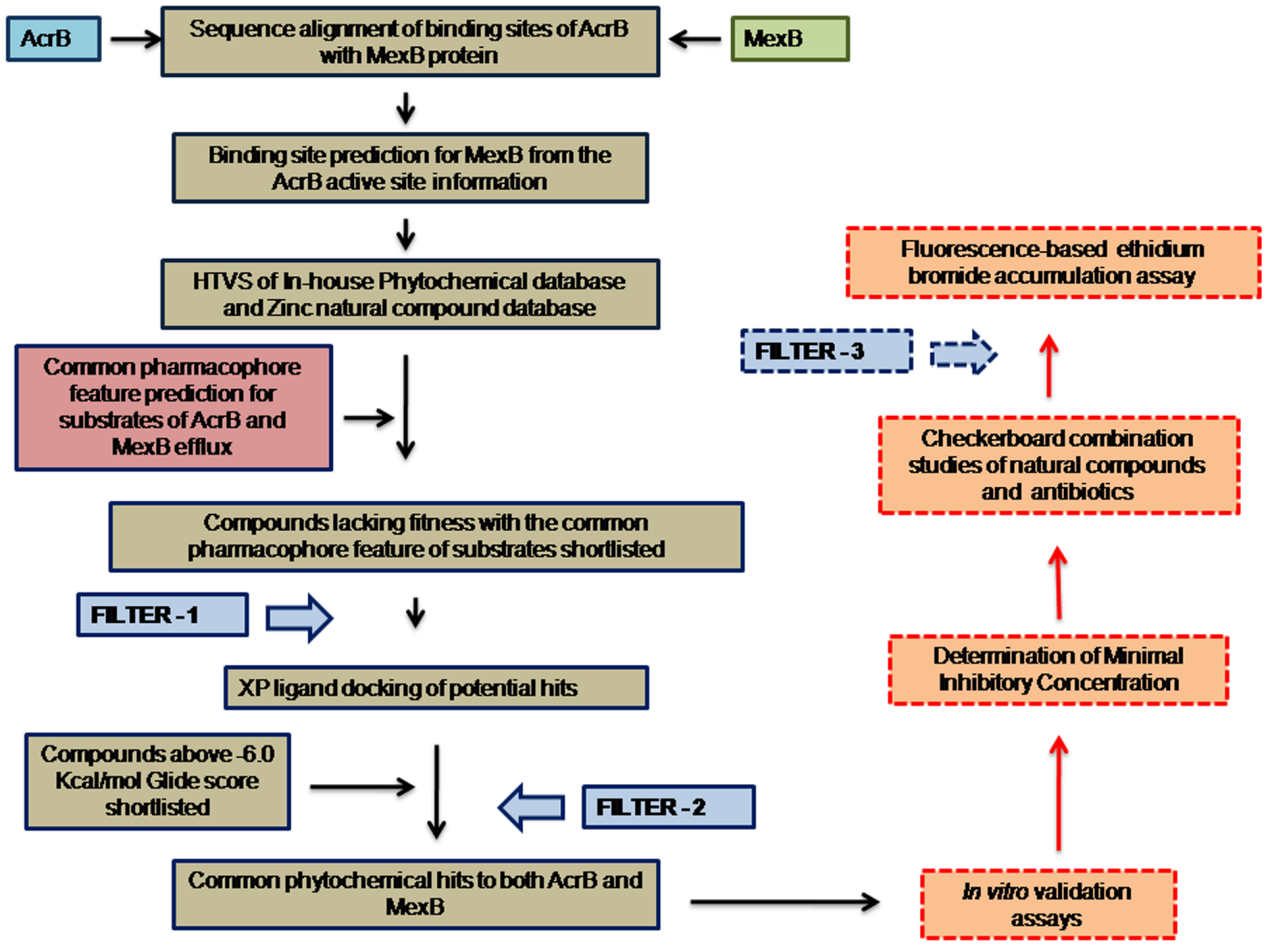
Flow chart of Virtual screening, pharmacophore-based filtering and experimental screening strategy for identifying efflux inhibitors.

In this study, we have focused on overcoming the drug resistance in *P. aeruginosa* and *E. coli* by identifying natural compounds from plants that can bind and have effect on integral membrane proteins MexB and its counterpart AcrB by *in silico* virtual screening and pharmacophore approach. *In silico* virtual screening and pharmacophore filtering approach are computational methods that select a subset of compounds from a database by predicting their binding mode against a target protein. The virtual screening was carried out based on ligand docking and their interactions with amino acid residues in two MC-207110 binding sites of the target proteins AcrB and MexB. The natural compounds shortlisted based on their docking scores were subjected to a reverse selection approach of pharmacophore screening. The hits shortlisted using pharmacophore approach was taken for XP ligand docking and free binding energy calculations. The refined top hit phytochemicals were taken for *in vitro* validation. The efficacy of the identified compounds in potentiating the activity of the antibiotics of *P. aeruginosa* MexAB-OprM over-producing strain and *E. coli* AcrAB-TolC over-producing strain was studied using checkerboard synergy assay and fluorescence based ethidium bromide accumulation bioassay. The workflow for the study is given as in **Figure 1**.

## Materials and Methods

### Chemicals, strain and growth media

Natural compounds – diadzein, lanatoside C, protocatechuic acid, scopolamine, gentisic acid, umbelliferone and MC-207,110 were purchased from Sigma-Aldrich (India). Cyanide-m-chlorophenylhydrazone (CCCP), ethidium bromide, carbenicillin and levofloxacin were purchased from Hi-Media, India. Greiner Bio-one flat bottomed black 96-well plate was purchased from Sigma-Aldrich. *P. aeruginosa* MexAB-OprM harbouring strain was kindly provided as a gift by Dr. Keith Poole, Queen's University, Canada and *E. coli* AcrAB-TolC harbouring strain was kindly provided as a gift by Dr. Klaas Martinus Pos, Goethe University, Germany. MIC and checkerboard assays were performed in the Cation adjusted Mueller Hinton Broth (CA-MHB) purchased from Hi-Media, India. Luria-Bertani (LB) broth purchased from Hi-media, India was used for ethidium bromide accumulation studies.

### *In silico* screening

#### Protein preparation

The MexB (PDB: 2V50) and AcrB (PDB: 1T9Y) protein structures were retrieved from Protein Data Bank. The MexB and AcrB crystal structures were prepared for the *in silico* studies using protein preparation wizard module (Epik version 2.2; Impact version 5.7; Prime version 3.0) of Schrödinger Suite [16] that prepares the protein by fixing errors in the protein like incomplete residues or missing residues near the active site. The module also adjusts the ionization and tautomerization state of protein, refines the structure by relieving any strain from the adjustments.

H-bond assignment was followed by H-bond optimization using “standard” mode of Prot-Assign algorithm. Restrained minimization of the protein was done using Impref module of Impact with a force field of OPLS_2005 [16], where all-atom minimization was done with termination based on convergence or reaching a heavy atom RMSD of 0.30 Å. We have used the default parameters such as deleting waters beyond 5 Å from the het group during pre-processing of the protein and removal of waters with less than three hydrogen bonds to non-waters before minimization.

#### Site map prediction for MexB protein

Prediction of binding cavity was done for the MexB 2V50 protein structure using SiteMap version 2.6. The potential ligand binding sites are identified by linking together “site points” that are close to the protein surface and protected from the solvent. The SiteScore, the scoring function, helps in accurately ranking possible binding sites and in eliminating sites that are not likely to be of pharmaceutical relevance [16]. SiteMap predicts five potential binding cavities in a protein and each cavity is assigned a score and ranked accordingly. The binding site with highest site score was taken for docking studies.

#### Receptor Grid Generation

**Figure 2:**
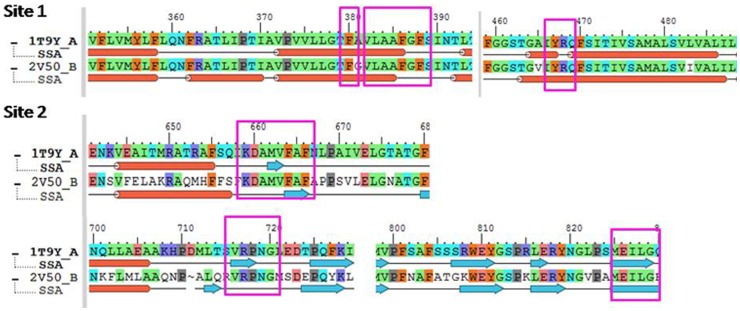
Alignment of protein sequences of MexB and AcrB. Sequences in box are the conserved residues in the binding site of MC-207110 in AcrB and MexB.

Receptor Grid Generation (OPLS_2005 force field) panel was used to specify the grid. With the absence of a inhibitor-MexB co-crystal PDB structure of *P. aeruginosa*, AcrB- MC-207110 complex structure (PDB ID: 1T9Y) from *E. coli* was used to identify the binding site information for *P. aeruginosa* MexB. Since a structural correlation (RMSD of 1.4 Å) exists between MexB of *P. aeruginosa* and AcrB of *E. coli* integral membrane proteins, the MC-207110 binding regions of AcrB were mapped on MexB [17]. Two MC-207110 molecules were bound to AcrB structure at two different sites. Pair-wise sequence alignment was performed using Prime module version 3.0 [16] for AcrB and MexB protein sequences. The comparison of the binding sites of MC-207110 of AcrB with MexB structure was done and the common amino acid residues at the binding site of MC-207110 were selected and used for Grid generation (Figure 2). Site 1 (MC-207110 molecule number 7001) corresponds to Ala385, Phe386, Gly387, Phe388 and Arg468 having hydrophobic contact with MC-207110. Site 2 (MC-207110 molecule number 7002) corresponds to the binding of MC-207110 with Phe664, Val716, Arg717, Pro718, Leu828 and Gly829 of AcrB protein.

#### Natural compound database

The information of the natural compounds with known antimicrobial and anticancer bioactivity information from plants was obtained from Dr. Duke's Phytochemical and Ethnobotanical Database and their structures were retrieved from Pubchem database and subjected for ligand preparation. The LigPrep module version 2.5 [16] prepares high quality, all-atom 3D structures for natural compounds in the database.

The module generates single, low-energy, 3D coordinates with correct chiralities for each input structure. Ionization states for the compounds were generated at pH of 7.0±2.0 with Epik and tautomeric states were generated and the ligands were desalted. The Epik state penalty is computed in units of Kcal/mol, thereby making it directly compatible with the GlideScore used for docking [18]. A force field of OPLS_2005 was used for the minimization of the compounds. A maximum of 32 stereoisomers were generated for each ligand with about 100 low energy ring conformations per ligand.

### High throughput virtual screening (HTVS) of phytochemicals against the AcrB and MexB integral membrane proteins

The virtual screening workflow of Maestro 9.2 version [16] was used to screen the collections of natural compounds against the two MC-207110 binding sites of AcrB and MexB protein targets. The virtual screening workflow offers selective filtration of ligands with increased strictness based on their efficiency to interact with the binding cavity residues.

The virtual screening workflow includes ligand preparation using LigPrep as an initial step; since a prior Ligprep was performed for the database this step was skipped. The receptor grid file was added and screening based on docking was performed with defaults parameters like use of Epik state penalties for docking. The docking accuracy level was set only for High throughput virtual screening (HTVS) for screening the natural compound database. The scaling factor was kept at default of 0.8 and partial charge cut-off was kept at 0.15. Force field of OPLS_2005 was used during the docking process. By default virtual screening workflow module retains 10% of the best compounds for both MexB and AcrB target proteins [16]. The shortlisted hits were used for pharmacophore based screening for filtering out the compounds having features similar to the antibiotic substrates that are effluxed out of the bacterial cell.

### Common pharmacophore model of efflux substrates

Experimental inhibitory activity data were obtained from literature and compiled in the database of substrates [5]. The structures of the substrates were retrieved from drug bank repository and were converted to low energy structures using LigPrep script version 2.5 [16]. Common pharmacophore model was developed using 49 substrates, that are effluxed out of MexB and AcrB using Phase module version 3.3 [16]. Conformers were generated for the substrates by using Monte Carlo plus Minimization (MCMM)/Low Mode Search (LMOD) method [16] with a maximum of 2,000 steps with a distance-dependent dielectric solvent model and an OPLS-2005 force field. Default set of six pharmacophore features that includes hydrogen bond acceptor (A), hydrogen bond donor (D), hydrophobic group (H), negatively charged group (N), positively charged group (P) and aromatic ring (R) were defined by three chemical structure patterns, namely point, vector, and group as SMARTS queries. Common pharmacophore features represent generalized molecular features that include the above mentioned 3D aspects responsible for their biological activity. The pharmacophore models of substrates were studied for their different spatial arrangements and ranked using survival score. The hypothesis with a high survival score exhibits the high average similarity to other members of its cluster. The survival score [16] is defined as below. 
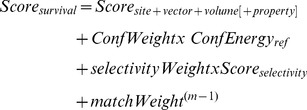
(1)


*ConfEnergyref* is the reference ligand relative conformational energy, *Score* is an empirical estimate of the rarity of the hypothesis, *confWeight*, *selectivityWeight* and *matchWeight* are user-adjustable parameters and *m* is the number of ligands in the alignment

The phytochemicals shortlisted using virtual screening was screened against all the pharmacophore models generated for the substrates and filtered based on their fitness against the pharmacophore models. Scoring function [16] for fitness with respect to actives was calculated using default parameters for site, vector, and volume and defined as:
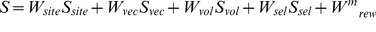
(2)


The hits obtained having maximum fitness of substrate pharmacophore model were not included for the further studies (reverse selection approach) and compounds lacking fitness were taken for Glide XP ligand docking calculations.

### Glide XP-ligand docking

Glide XP ligand docking approach was performed using Glide (Grid-based ligand docking from energetics) program of Schrodinger suite [16]. The XP docking helps in removing the false positives and the scoring function is much stricter than the HTVS [19]. It uses the Chemscore function for scoring the hits. The docking and scoring function were validated before the docking was performed. This was done by re-docking the MC-207,110 molecule in the binding regions of AcrB protein with an root mean square deviation (RMSD) of 0.910 Å between the co-crystal structure and the redocked conformation, this re-docking increases the precision and accuracy of the selected binding cavity. All the hits lacking good fit with the pharmacophore model of efflux substrates, presumed to be less susceptible to efflux pumps were docked against two different binding sites in the structures of AcrB and MexB. A multi-ligand scoring function of Schrödinger's, GlideScore (GScore) was used to rank the ligand poses of the docked complex. GlideScore devised by Schrödinger [16] is given as
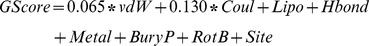
(3)where, vdw denotes the Van der Waals energy; Coul, Coulomb energy; Lipo, Lipophilic term derived from hydrophobic grid potential; HBond, Hydrogen-bonding term; Metal, Metal-binding term; BuryP, penalty for buried polar groups; RotB, penalty for freezing rotatable bonds; Site, polar interactions in the active site.

The greater the XP Glide score, the better affinity of the hit to bind to the protein target. Further, the protein and hit interactions were analyzed and compared with the interactions of known inhibitor MC-207110 for screening hits that have interactions with active site amino acids.

### Molecular mechanics/generalized born surface area (MM-GBSA) calculations of shortlisted phytochemicals

All the docking studies are not successful; a further more accurate computational method is required for lead hit identification. MM-GBSA calculates binding free energies for the best hit docked complexes using MM force fields and implicit solvation. This method is very useful in computing the relative binding affinities that each hit requires for the target protein [20]. This method is used as a filter to shortlist the ligands for *in vitro* validation. The Prime module version 3.0 [16] was used to determine the binding free energies of the docked complexes. This protocol uses MM-GBSA technology for finding the different binding energies of the complex, ligand and receptor. The pose viewer file was used to generate the binding energy calculations. All protein atoms were rigid, and only the ligand structure was relaxed by default. The binding energy [16] was calculated based on the following equation. The protein-ligand complexes were ranked based on their free energy calculation. 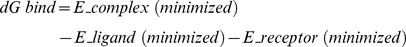
(4)


### *In vitro* validation assays

#### In vitro checkerboard synergy assay

Microplate dilution was done to determine the minimal inhibitory concentration (MIC) of the drugs and compounds as per Clinical and Laboratory Standards Institute (CLSI) guidelines [21]. The checkerboard synergy titration assay of antibiotics in combination with the known inhibitor and the test compounds was performed in 96-well microtitre plate. The range of concentrations tested for each antimicrobial agent was eight dilutions lower than the MIC and two dilutions higher than the MIC. A final concentration of 4x MIC of the drug (50 µl) was added in the last well (column 12) of the 96-well checkerboard panel for each antibiotic. The test compounds were serially diluted at different sub-inhibitory concentrations and 50 µl was added to each well. The bacterial strains were adjusted to 10^6^ cfu/ml cell density and 50 µl was used in each well. The fractional inhibitory concentration (FIC) index of compound was calculated as given by the formula. 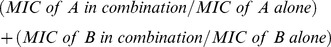
(5)


A FIC≤0.5 for a compound was taken as synergistic, FIC>0.5–4 was additive/indifferent and FIC>4 was taken as antagonistic.

#### Ethidium bromide accumulation assay

The ability to accumulate ethidium bromide by the bacterial strains, studied with and without the presence of the test phytochemicals was done as described previously [22]. Overnight culture of the bacterial strains were inoculated in LB broth and incubated for about 4 hr at 37°C. The bacterial cells were centrifuged at 10,000 rpm for 10 min and the pellet was resuspended in phosphate buffered saline (PBS) and adjusted to OD of 0.1 at 600 nm. Culture, 180 µl, was added to each well of the flat bottomed, black 96-well plate. PBS was used as blank and 10 µl of ethidium bromide was added to each well to give a final concentration of 2.5 µM. CCCP, an inhibitor of efflux pump and the test compounds were added before the plate was read. The plate was incubated at 37°C and the top reading was taken at an excitation of 530 nm and emission of 590 nm wavelengths for 30 minutes with a 5 minute interval between each cycle using BioTek Synergy microplate reader. Each test was repeated thrice and all results were statistically analyzed. The loss of ethidium bromide from the cells was indicated by the reduction in the fluorescence as compared to the control tube without any test compounds. The ability of the compounds to accumulate ethidium bromide was thus defined based on the percentage increase in fluorescence. The percentage increase in fluorescence [23] was calculated as below: 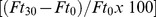
(6)


Where, Ft_30_ is the ethidium bromide fluorescence at time 30 min and Ft_0_ is fluorescence at time 0 min.

## Results

### High-throughput virtual screening of phytochemicals against MexB and AcrB

In the present study two MC-207110 binding sites (site 1 and site 2) in the AcrB (PDB 1T9Y) protein and their corresponding regions in the MexB (PDB 2V50) protein were taken as active sites for HTVS of the phytochemicals were selected.

The MC-207,110 molecule binds at two regions in the AcrB structure: At binding site 1, the participation of the residues, Phe664, Arg717, Pro718, Leu828, Ser715 were involved and at the central cavity (binding site 2) residues Phe386, Phe388 and Phe459 were involved in MC-207110 binding. The binding site residues of AcrB were conserved among the MexB protein sequence as determined by Prime sequence alignment. To confirm the results of the binding site obtained from the sequence alignment, the binding site of MexB protein was also predicted using Site map tool. The tool predicts five potential binding sites and the MC-207110 binding site falls into the top scoring site in the MexB protein with a SiteScore value of 1.079. Hence by this study the active site in MexB protein was deduced and was used for identifying the inhibitors from Dr. Duke's phytochemical and ethnobotanical database.

A total of 70 phytochemicals passed the HTVS for the site 1 and 109 for site 2 when screened against AcrB with a maximum Glide score of −8.05 Kcal/mol for site 1 and −7.79 Kcal/mol for site 2. For the MexB counterpart, lesser number of compounds was shortlisted, 36 phytochemicals were shortlisted against the site 1 with maximum Glide score of −6.93 Kcal/mol and 48 against site 2 with −6.99 Kcal/mol as the maximum score. The shortlisted HTVS hits were further refined and filtered by common pharmacophore based screening and XP ligand docking.

### Common pharmacophore model of efflux substrates

A total of 49 substrates, common to both AcrB and MexB efflux pump systems, were selected to create a common pharmacophore model. The pMIC values were calculated from the MIC of the efflux substrates collected from literature. Active and inactive thresholds of pMIC were set as 9.88 and 5.08 respectively, which were applied to the dataset of the 49 substrates. The threshold yielded 38 actives and 11 inactives based on pMIC, that were used for common pharmacophore model generation and subsequent scoring. The common pharmacophore hypotheses generated for the 49 substrates are the probable features that make them more likely to get effluxed out of the cell. Since antibiotic efflux is the main mechanism in multidrug resistance in Gram-negative bacteria, the features generated from the above substrates were used to filter the compounds. A total of 83 probable five-featured common pharmacophore hypotheses belonging to 11 types (AADHR, ADHNR, AAHNR, AADHN, AADNR, AAADN, AAADR, AAANR, AAAHN, AAADD and AAADH) were subjected to scoring function. The generated hypotheses with their inter-site distances are shown in **Table S1**. The 263 hits shortlisted from the HTVS were screened against the common pharmacophore model of the substrates. A reverse filtering approach was applied, where the hits with maximum fitness to one or more of the substrate pharmacophore models were excluded from further study. Hence the phytochemicals that were filtered using the pharmacophore based approach have a better possibility of being active inhibitors *in vitro*. A total of 63 (23.95%) phytochemicals among the HTVS hits had good fit value against the pharmacophore model of the efflux substrates. About 200 (76.04%) hits that lacked fitness with the model were further subjected for binding affinity calculations using the XP ligand docking approach.

### XP glide ligand docking and MM-GBSA calculations of shortlisted phytochemical hits against AcrB and MexB protein targets

The 200 compounds filtered using pharmacophore based approach were docked against the two binding sites of AcrB and MexB proteins. The compounds were sorted based on their XP Glide score and their interactions with the active site amino acids in the binding cavity. Since AcrB and MexB have high structural similarity and same inhibitor binding sites, the compounds that repeated in occurrence among both AcrB and MexB at the binding sites were considered for the *in vitro* synergy tests. **Table 1** shows the interactions, score of MC-207110 and the repeated compounds shortlisted for site 1 both in AcrB and MexB proteins; **Table 2** for site 2 both in AcrB and MexB proteins. The interactions and the binding modes of the shortlisted compounds with the active site residues of AcrB and MexB protein are discussed below.

**Table 1:** Molecular interactions of phytochemicals with the active site residues in the binding site 1 of the target proteins, MexB of *P. aeruginosa* and AcrB of *E. coli*.

	Phytochemical (Pubchem ID)	H-bond	Hydrophobic contacts	Glide XP Gscore (Kcal/mol)	Glide energy (Kcal/mol)	MM-GBSA (Kcal/mol)
**MexB Site 1**	MC-207,110	N—H—O(Gly387) N—H—O(Ty35) N—H—O(Asn135)	Ser389, Gly296, Ala384, Pro31, Pro36, Ala37, Gly97, Arg468, Phe388, Gln469	−5.57	−56.26	−34.67
	Lanatoside-C (3879)	(Thr295)N—H—O (Leu293)O—H—O O—H—O(Gly387)	Pro30,Pro36, Gln469, Ala384, Phe388, Gly296, Ala385, Thr295, Gly381	−8.70	−65.61	−102.55
	Protocatechuic-acid (72)	O—H—O (Gln469) (Ser389)N—H—O	Asn391, Pro36,Phe388, Asn33, Ala37	−5.65	−21.28	−24.24
	Gentisic-acid (3469)	O—H—O (Gln469) (Gln469)N—H—O	Arg468,Ser389, Asn391, Ala37, Pro36,Phe388, Val465	−4.38	−19.89	−22.12
	Diadzein (5281708)	(Ser389)N—H—O (Gly296)N—H—O	Ser389, Gly387, Gln469, Arg468, Thr295, Asn391, Ala37	−4.37	−32.36	−36.14
**AcrB Site 1**	MC-207,110	N—H—O (Gln469) (Ala297)N—H—O O—H—N(Ala297) N—H—O(Gly387) N—H—O(Tyr35)	Ser389, Asn391, Thr37, Gly296, Phe388, Ala465, Asn298	−6.72	−56.59	−54.78
	Lanatoside-C (3879)	O—H—O(Ala385) (Arg468)N—H—O (Ser389)N—H—O O—H—O (Gly296) (Asn298)N—H—O	Phe386,Gly2387, Phe388, Gln469, Asn391, Ala384, Leu30	−6.76	−56.62	−62.91
	Gentisic-acid (3469)	O—H—O (Gln469) (Arg468)N—H—O (Arg468)N—H—O	Phe388, Thr37, Ser389, Ala465, Pro36	−4.68	−21.00	−24.39
	Diadzein (5281708)	O—H—O(Ala457)	Val454,Phe386,Phe388, Ile472, Phe459, Arg468	−3.76	−26.37	−38.71

**Table 2:** Molecular interactions of phytochemicals with the active site residues in the binding site 2 of target proteins, MexB of *P. aeruginosa* and AcrB of *E. coli*.

	Phytochemical (Pubchem ID)	H-bond	Hydrophobic contacts	Glide XP Gscore (Kcal/mol)	Glide energy (Kcal/mol)	MM-GBSA (Kcal/mol)
**MexB Site 2**	MC-207,110	N—H—(Asn718) N—H—(Glu816) N—H—(Glu825) N—H—(Phe617)	Lys134, Arg716, Phe666, Gly828, Pro717, Phe136, Phe573, Lys814, Gly719	−8.39	−48.74	−57.23
	Lanatoside-C (3879)	(Arg714)N—H—O O—H—O (Asn676) (Gln575)N—H—O O—H—O (Asn718) (Ser721)O—H—O	Phe664,Arg716, Gly719, Ser721, Phe617, Asn718, Pro717, Leu827	−8.74	−58.12	−30.76
	Scopolamine (5184)	O—H—O (Asn676)	Gln575, Asn718, Leu827, Lys134, Phe617	−6.19	−31.524	−29.53
	Umbelliferone (5281426)	(Gly719)O—H—O (Gln575)N—H—O	Phe664, Ala618, Met720, Arg716, Asn718	−3.20	−23.95	−53.46
**AcrB Site 2**	MC-207,110	N—H—O(Ser134) N—H—O(Glu826) N—H—O(Glu826) N—H—O(Asp681) N—H—O(Glu673) N—H—O(Glu673)	Leu828, Phe664, Phe666, Phe617, Val672, Asn719	−7.48	−58.68	−42.67
	Lanatoside-C (3879)	O—H—O (Ala831) O—H—O (Gln830) (Gln657)	Phe617, Phe664, Arg716, Pro717, Leu827	−9.12	−60.744	−88.50
	Scopolamine (5184)	O—H—O(Ala665)	Lys134, Gln575, Ala618, Phe617, Arg716, Asn718, Leu827	−4.19	−34.05	−38.64
	Umbelliferone (5281426)	(Gln675)N—H—O O—H—O(Gly789)	Ala618, Phe664, Arg716, Pro717, Asn718	−3.72	−19.279	−40.80

#### Interactions of phytochemicals in Site 1 of MexB and AcrB

**Figure 3:**
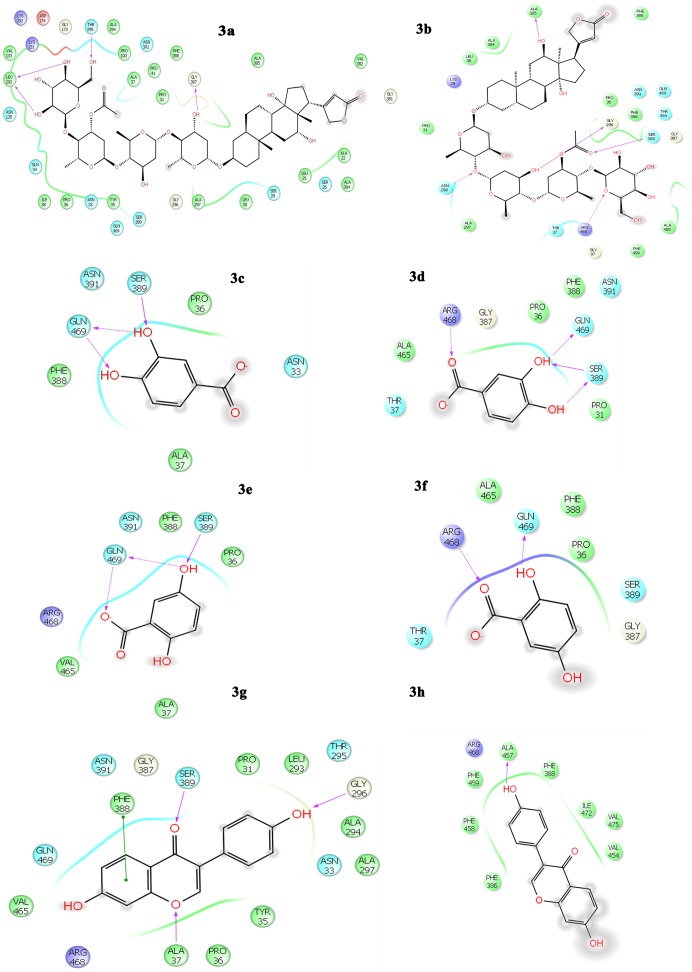
Interaction of selected phytochemicals with the active site residues in the binding site 1 of the integral membrane proteins MexB of *P. aeruginosa* and AcrB of *E. coli*. Interaction with MexB of *P. aeruginosa*: 3a - lanatoside C, 3c - protocatechuic acid, 3e - gentisic acid, 3g – diadzein; Interaction with AcrB of *E. coli*: 3b - lanatoside C, 3d - Protocatechuic acid, 3f - gentisic acid, 3h - diadzein.

Lanatoside C interacted with the residues in close proximity to the binding domain of the MexB and AcrB protein. The hydroxyl groups of the glucose moiety of lanatoside C interacted with Leu293, Thr295 of MexB and Arg468 of AcrB protein. The digitoxose sugar moiety of lanatoside C formed H-bond with Gly387 of MexB and Ser389, Gly296, Asn298 of AcrB (**Figure 3a and 3b**). Protocatechuic shared H-bonds with Gln469, Ser389 of both MexB and AcrB. With AcrB, the complex is additionally stabilized with two H-bonds with Arg468 (**Figure 3c and 3d**). Gentisic acid formed H-bond with Gln469 with MexB and AcrB and there is a conformational change in the compound that led to the formation of another H-bond with Arg468 of AcrB (**Figure 3e and 3f**). With MexB, diadzein formed H-bonds with Gly296 Ser389, a π-π stacking with Phe388 and a single H-bond with Ala457 of AcrB (**Figure 3g and 3h**).

#### Interactions of phytochemicals in Site 2 of MexB and AcrB

**Figure 4:**
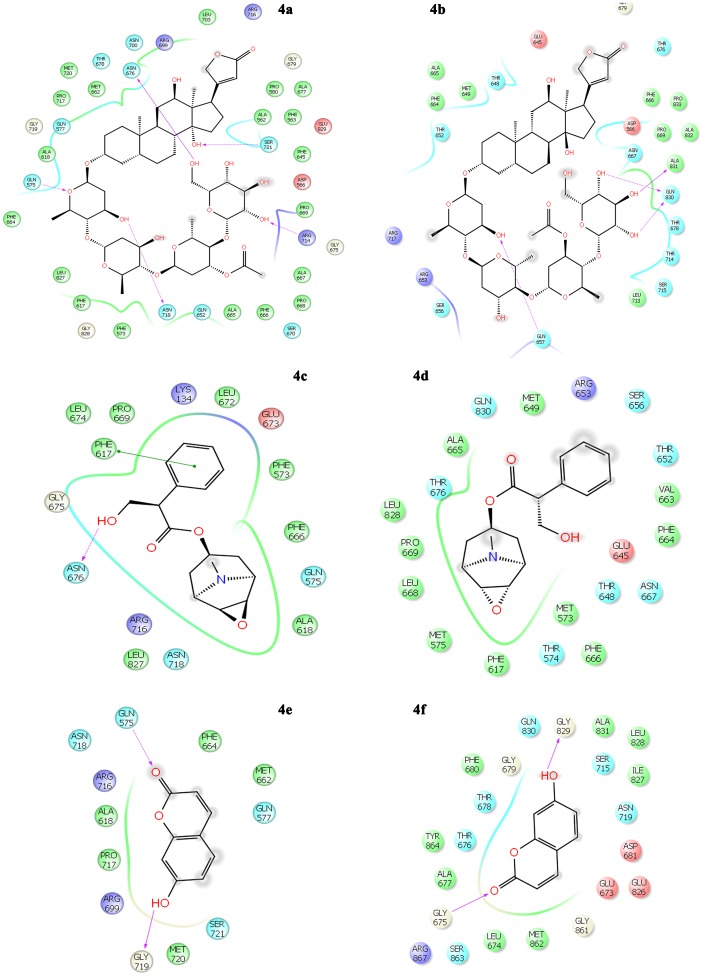
Interaction of selected phytochemicals with the active site residues in the binding site 2 of the integral membrane proteins MexB of *P. aeruginosa* and AcrB of *E. coli*. Interaction with MexB of *P. aeruginosa*: 4a - lanatoside C, 4c – scopolamine, 4e – umbelliferone; Interaction with AcrB of *E. coli*: 4b – lanatoside C, 4d – scopolamine, 4f - umbelliferone.

The glucose moiety of lanatoside C formed two H-bonds with Asn676 and Arg714 of MexB and Ala831 and Gln830 of AcrB. The digitoxose sugar moiety formed H-bond with Asn718 of MexB and Gln657 of AcrB (**Figure 4a and 4b**). Scopolamine formed a single H-bond with Asn676, a π- π stacking with Phe617 with MexB and Ala665 of AcrB (**Figure 4c and 4d**). Umbelliferone shared hydrogen bond with Gly719, Gln575 and with AcrB, there was a shift in the ligand position with interactions to Gly675 and Gly829 (**Figure 4e and 4f**).

The final ranking of protein-ligand complex for both MexB and AcrB based on the binding affinities was done using Prime MM-GBSA method. The compounds were ranked according to their free energy estimates. Lanatoside C had the least binding free energy MM-GBSA dG bind of −102.55 and −30.76 Kcal/mol with MexB site 1 and site 2 respectively. With AcrB complex, lanatoside C had energy of −62.91 and −88.506 in site 1 and site 2 respectively. Scopolamine had the second least energy with site 2 and diadzein with site 1 (**Table 1** **and** **Table 2**).

### *In vitro* checkerboard synergy assay

The combination effect of the antibiotic and the inhibitors was studied by using the checkerboard microtitre assay. The MIC of antibiotics carbenicillin, levofloxacin and natural compounds were identified as per CLSI guidelines (data not shown). The EPI, MC-207110 was used as a control for positive synergistic effect. The interactions between different compounds with antibiotics are classified as synergistic or indifferent/additive as given in **Table 3**.

**Table 3:** Effect of phytochemicals and known EPI on the MIC of antibiotics using checkerboard synergy assay in *P. aeruginosa* and *E. coli*.

Antibiotics + phytochemicals^a^	FIC index	Interpretation
***P. aeruginosa*** ** MexAB-OprM**		
C+ MC-207110	0.04	Synergy
L+ MC-207110	0.05	Synergy
C+ Lanatoside C	0.14	Synergy
L+ Lanatoside C	0.08	Synergy
C+ Diadzein	0.26	Synergy
L+ Diadzein	0.27	Synergy
C+ Gentisic acid	0.51	Indifferent
L+ Gentisic acid	1.02	Indifferent
C+ Protocatechuic acid	0.51	Indifferent
L+ Protocatechuic acid	0.27	Synergy
C+ Scopolamine	0.52	Indifferent
L+ Scopolamine	1.01	Indifferent
C+ Umbelliferone	0.51	Indifferent
L+ Umbelliferone	0.52	Indifferent
***E. coli*** ** AcrAB-TolC**		
C+ MC-207110	0.03	Synergy
L+ MC-207110	0.04	Synergy
C+ Lanatoside C	0.25	Synergy
L+ Lanatoside C	0.13	Synergy
C+ Diadzein	0.25	Synergy
L+ Diadzein	0.13	Synergy
C+ Gentisic acid	1	Indifferent
L+ Gentisic acid	0.51	Indifferent
C+ Protocatechuic acid	0.25	Synergy
L+ Protocatechuic acid	0.51	Indifferent
C+ Scopolamine	0.5	Synergy
L+ Scopolamine	1.01	Indifferent
C+ Umbelliferone	1	Indifferent
L+ Umbelliferone	0.51	Indifferent

FIC index - ≤0.5: synergistic, >0.5–4: indifferent and >4: antagonistic.

a Sub-MIC concentrations: MC-207110 - 8 mg/L; Lanatoside C 16 mg/L; Diadzein 16 mg/L; Gentisic acid 16 mg/L; Protocatechuic acid 16 mg/L; Scopolamine 16 mg/L; Umbelliferone 16 mg/L.

Six natural compounds shortlisted from the *in silico* HTVS and pharmacophore analyses were screened *in vitro* using the synergy test. The interaction of MC-207110 was synergistic with the antibiotics tested against *E. coli* and *P. aeruginosa*. Synergistic effect of MC-207110 (8 mg/L) on carbenicillin and levofloxacin in *E. coli* was with an FIC index of 0.03 and 0.04 respectively. The synergy for MC-207110 with carbenicillin and levofloxacin against *P. aeruginosa* was with FIC index 0.04 and 0.05 respectively indicating a synergistic effect. Gentisic acid and umbelliferone did not have any significant synergistic effect on the antibiotics. Protocatechuic acid exhibited synergy with levofloxacin in *P. aeruginosa* and with carbenicillin in *E. coli*. Scopolamine showed synergy only with carbenicillin in *E. coli*.

Lanatoside C (16 mg/L) exhibited highly significant synergistic/potentiating effect in combination with both the antibiotics tested. Higher synergistic effect was observed with levofloxacin with a FIC index of 0.08 against *P. aeruginosa* and 0.13 for *E. coli*. The combination with carbenicillin also produced a synergistic interaction, with a FIC index of 0.14 for *P. aeruginosa* and 0.25 for *E. coli*.

Diadzein (16 mg/L) displayed a synergistic effect equal to that of Lanatoside C. In *P. aeruginosa*, the combination of diadzein with carbenicillin or levofloxacin gave similar interaction as that of Lanatoside C with FIC index of 0.26 and 0.27 respectively. In *E. coli*, the potentiating effect on levofloxacin was high with FIC index of 0.13 followed by 0.25 FIC index for carbenicillin.

### Ethidium bromide accumulation assay

**Figure 5:**
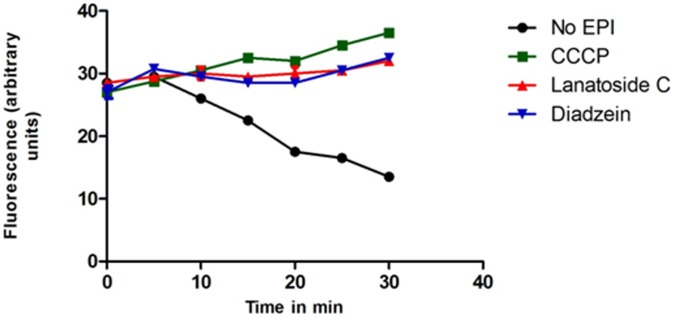
Effect of efflux inhibitor, CCCP and phytochemicals in accumulation of ethidium bromide in *P. aeruginosa* strain.

Since MC-207110 does not have any effect on the accumulation of ethidium bromide, an efflux pump substrate, the effect of CCCP on the accumulation was used in this study as a positive control. The compounds with positive synergistic effect were confirmed with the ability to accumulate ethidium bromide [8] [24]. **Figure 5** **and** **6** displays the fluorescence intensities observed with treatment of lanatoside C (16 mg/L) and diadzein (16 mg/L) and CCCP (8 mg/L) in *P. aeruginosa* and *E. coli* respectively against a time period. The fluorescence intensity was the least for the well with no EPI and maximum for the well with positive control, CCCP. The treatment with lanatoside C and diadzein induced a comparatively higher accumulation of ethidium bromide within the cell as exhibited by the increase in fluorescence intensity following their addition against both *P. aeruginosa* and *E. coli* cells. The percentage of increase in fluorescence of ethidium bromide in the presence of CCCP was 32.72% and 35.18% in *E. coli* and *P. aeruginosa* respectively. Among the two compounds tested, the presence of diadzein had a better percentage of increase in fluorescence with an accumulation of 16.36% and 20.37%; while for lanatoside C it was 14.28% and 12.28% accumulation in *E. coli* and *P. aeruginosa* respectively. The increase in the fluorescence intensities of ethidium bromide (increased accumulation) by the natural compounds correlated with their ability to increase the MIC of the antibiotics when studied using checkerboard synergy tests.

**Figure 6:**
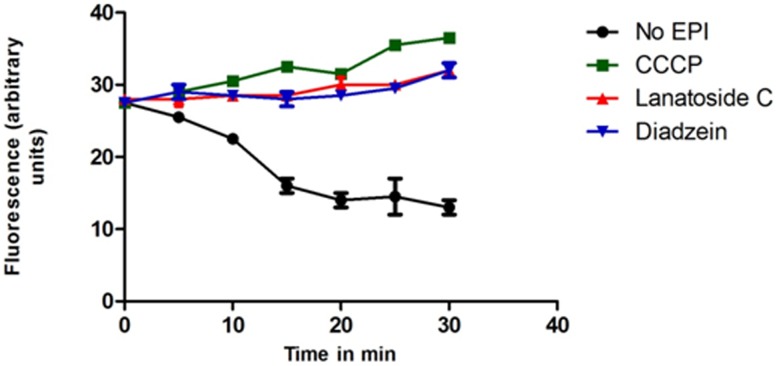
Effect of efflux inhibitor, CCCP and phytochemicals in accumulation of ethidium bromide in *E. coli* strain.

## Discussion

Multidrug efflux proteins are involved as the resistance mechanism for many Gram-negative bacteria against wide classes of antibiotics. The reversal of this resistance, *via* inhibition of drug efflux mechanisms, is a promising research area. *P. aeruginosa* and *E. coli* are among the most commonly encountered resistant bacteria in the clinical settings. Improving or changing the chemical design of the antibiotics is a means to fight drug resistance; but the organisms are capable of overcoming the change very soon. A study by Chung *et al*., (2005) on the activity of flavonoids against the cancer efflux pump, p-glycoprotein has shown that the potentiating activity of quercetin was almost similar to that of the known p-glycoprotein inhibitor [25]. With previous evidence of natural products as EPIs on cancer efflux and also against pumps in the Gram-positive bacteria, we have screened natural compounds from various plants using *in silico* high-throughput virtual screening and common pharmacophore based approach and validated by *in vitro* assay.

AcrB and MexB are the integral membrane proteins of the AcrAB-TolC and MexAB-OprM efflux system belonging to *E. coli* and *P. aeruginosa* respectively. In this study we have identified inhibitors against these two integral membrane proteins using the crystal structures 2V50 for MexB and 1T9Y for AcrB. MC-207110 acts as a competitive inhibitor for MexB and AcrB efflux systems and binds to the integral membrane protein as given in the crystal structure of AcrB [26]. Using sequence alignment and binding site prediction the binding sites for MexB protein was conferred. The predicted binding site had a SiteScore of above 1, suggesting that the site is of particular promise in drug binding [16].

A recent structure for MexB with inhibitor co-crystallized with AcrAB/MexAB specific inhibitor of pryridopyrimidine derivative (ABI-PP) was solved at a resolution of 3.15 Å (PDB 3W9J) by Nakashima *et al.*, 2013. The ABI-PP binding site in MexB is similar to the site in AcrB. Based on the crystal structure information from 3W9J, the hydrophobic tert-butyl thiazolyl aminocarboxyl pyridopyrimidine moiety of ABI-PP was inserted into the narrow hydrophobic trap surrounded by Phe136, Phe178, Phe610, Phe615, Phe628 and Phe573. The hydrophilic tetrazole ring interacts with residues Asp274, Arg620 and Lys151 of MexB [27]. Though the binding site that was used in this study was not same as that of the binding site of ABI-PP (3W9J), they were in close proximity to each other and also fall under the single binding cavity predicted for MexB 2V50.

We employed HTVS docking option, to identify the novel hits from Dr. Duke's phytochemical and ethnobotanical database. A total of 179 phytochemicals were shortlisted using HTVS against AcrB and 84 compounds were shortlisted against MexB. The compounds (23.95%) among the HTVS screened hits shared similar features to the efflux substrates pharmacophore and hence have a higher probability of being effluxed themselves. The hits that shared minimal fitness (76.04%) with the efflux substrate model have less chance of being substrates of the AcrB and MexB efflux pumps. According to Peach and Nicklaus, a filtering method based on pharmacophore provides additional information to the docking calculations and scoring functions [28]. Another approach that could be applied for filtering is e-pharmacophore screening, where the hypothesis generated for a known inhibitor will be used to screen against database [29].

The Glide XP docking represents a sole, rational approach for docking where, the simultaneous optimization of sampling algorithms and the scoring function are carried out. XP method was tested with wide range of ligands and protein targets and has given improvement in binding affinities and database enrichment [30]. Before performing the docking the docking program was validated by redocking co-crystal ligand (RMSD 0.910 Å) to determine the reproducibility of docking program. Six compounds were ranked as top hits based on their scoring values and their similarity in the interaction with the AcrB and MexB active site residues when compared with MC-207,110. They were also evaluated based on their binding free energy calculations for further refined ranking. The phytochemicals, Lanatoside C, protocatechiuc acid, gentisic acid and diadzein interacted with the residues in close proximity to the binding site 1 of both MexB and AcrB protein. Compounds lanatoside C, scopolamine and umbelliferone shared bonded and non-bonded interaction with the residues in close proximity to the binding site 1 of both MexB and AcrB protein. Further the *in silico* results were validated using *in vitro* assay.

The checkerboard assay is used to study the combinations of antibiotics and potential EPIs as a screening method. The sub-inhibitory concentration of the natural compounds was used for the checkerboard synergy assay that gives the FIC index used to interpret the combinations. The synergistic effect of the known inhibitor MC-207,110 was compared with the phytochemicals. The best synergistic effect among all combinations tested with a six fold decrease in MIC was observed with combining lanatoside C and levofloxacin in *P. aeruginosa*. Protocatechuic acid exhibited synergy towards a single antibiotic combination against both the *E. coli* and *P aeruginosa* while scopolamine showed synergistic combination for a single drug against *E. coli*.

A fluorimetric study was employed to assess the fluorescence of ethidium bromide, a substrate of both the efflux pumps. The levels of accumulation of ethidium bromide within the *E. coli* and *P. aeruginosa* cells display the association of efflux pumps with the resistance mechanism. The fluorescence of ethidium bromide is maximal inside cells thereby the accumulation was evidenced by increase in the fluorescence units. The addition of the lanatoside c and diadzein showed slightly lesser accumulation of ethidium bromide in the cells than in the presence of CCCP; however the accumulation was significantly higher than that of the control without any EPI, in both *P. aeruginosa* and *E. coli*. Piperine showed potent inhibitory effect on *S. aureus* multidrug efflux pump NorA at a concentration of 50 mg/L [31]. However, the effect of lanatoside C and diadzein in inhibiting the MexB and AcrB efflux pumps was evident at a much lesser concentration.

The ability of plant-derived compounds to inhibit multidrug efflux pumps was explored previously by Gibbons *et al.*, 2007 [15]. Many phytochemicals have shown positive inhibitory effect on Gram-positive efflux pump proteins. Reserpine inhibited the Major Facilitator Superfamily and the ATP-binding cassette (ABC multidrug efflux) superfamily pumps [32]. Catechin gallates like epicatechin gallate had exhibited a weak inhibitory effect against the NorA efflux pump and Epigallocatechin gallate inhibited Tet(K) pumps in staphylococci [15]. Lanatoside C is a cardiac glycoside inhibiting the membrane bound Na^+^-K^+^-ATPase or sodium pump responsible for Na^+^-K^+^ exchange and increases intracellular Ca^2+^ concentration [33]. Potentiating effect of lanatoside C on the Gram-negative efflux systems, MexB and AcrB in this study could possibly be due to its inhibitory effect reported on sodium pumps. Diadzein, an isoflavone has also showed slight modulatory effect on *Mycobacterium smegmatis* as an EPI [34] and also increased the sensitivity of human cervical carcinoma KB-V1 cells over-expressing P-glycoprotein to drugs [35].

The ability to accumulate efflux substrate by the efflux pump inhibitors could increase the efficacy of antibiotics by raising internal cellular levels of the drug by reducing bacterial efflux mechanism [31]. Lanatoside C and diadzein are highly effective in potentiating the activity of the two antibiotics, carbenicillin and levofloxacin in efflux pump harbouring *P. aeruginosa* and *E. coli*.

## Conclusions

Molecular docking calculations accompanied by *in vitro* biological assay showed that lanatoside C and diadzein are possible inhibitors of the MexAB-OprM and AcrAB-TolC efflux pumps in *P. aeruginosa* and *E. coli*. The common pharmacophore hypotheses predicted for the substrates are probably the features that commonly occur among the diverse efflux substrates selected. The compounds lacking fitness are less likely to be effluxed, as they differ from the common pharmacophore feature model for the efflux substrates. The two compounds lanatoside C and diadzein shared a very low fitness with the common pharmacophore models predicted for the AcrB and MexB efflux substrates. Lanatoside C potentiated efficacy of two antibiotics tested against both *P. aeruginosa* and *E. coli* possibly by circumventing the efflux resistance mechanism. The addition of lanatoside C significantly increased the accumulation of ethidium bromide (14.28% for *E. coli* and 12.28% for *P. aeruginosa*) within the cell as evidenced by increase in fluorescence. Diadzein displayed a higher percentage of increase in fluorescence than lanatoside C with 16.36% and 20.37% for *E. coli* and *P. aeruginosa* respectively. Lanatoside C and diadzein had more or less similar potentiating effect on both carbenicillin and levofloxacin tested. Based on the potential to reduce MIC of antibiotics and to accumulate ethidium bromide in the fluorescence based assay, it is evident that lanatoside C and diadzein could have a higher possibility to counteract the MDR pumps in the resistant bacteria. Though, diadzein has shown EPI activity in bacteria, as per our knowledge there is no report on EPI activity of lanatoside C in Gram-negative efflux systems. As the incidence of drug resistant *P. aeruginosa* and *E. coli* are increasing in an alarming rate especially in the nosocomial patients, a combination therapy of antibiotics with these phytochemicals would be a better approach to combat the multidrug resistance. This will eventually help in better treatment outcome and help in decreasing the mortality rate of patients with infections caused by the MDR pathogens.

## Supporting Information

Table S1 Common pharmacophore hypotheses and model of the known efflux substrates of MexB and AcrB efflux systems.
(DOCX)
