# Supplementary material for: Identification of Natural Compound Inhibitors for Multidrug Efflux Pumps of Escherichia coli and Pseudomonas aeruginosa Using In Silico High-Throughput Virtual Screening and In Vitro Validation
Source: PLoS One. 2014 Jul 15;9(7):e101840. doi: 10.1371/journal.pone.0101840 (PMC4099075; doi:10.1371/journal.pone.0101840)
Supplement: Table S1 — Common pharmacophore hypotheses and model of the known efflux substrates of MexB and AcrB efflux systems. (DOCX) [file pone.0101840.s001.docx]

Identification of natural compound inhibitors for multidrug efflux pumps of *Escherichia coli* and *Pseudomonas aeruginosa* using *in silico* high-throughput virtual screening and *in vitro* validation

**Vasudevan Aparna^1^, Kesavan Dineshkumar^1^, Narasumani Mohanalakshmi^1†^, Devadasan Velmurugan^2^ and Waheeta Hopper^1^***

Vasudevan Aparna^1^

^1^Department of Bioinformatics, School of Bioengineering, Faculty of Engineering & Technology, SRM University, Kattankulathur - 603203,Tamilnadu, India

Kesavan Dineshkumar^1^

^1^Department of Bioinformatics, School of Bioengineering, Faculty of Engineering & Technology, SRM University, Kattankulathur - 603203,Tamilnadu, India

Narasumani Mohanalakshmi^1†^,

^1^Department of Bioinformatics, School of Bioengineering, Faculty of Engineering & Technology, SRM University, Kattankulathur - 603203,Tamilnadu, India

^†^Present address: Department of Biology, McGill University, Montreal, QC, Canada H3A 1B1

Devadasan Velmurugan^2^

^2^Centre of Advanced Study in Crystallography and Biophysics, University of Madras, Guindy Campus, Chennai - 600 025, Tamilnadu, India

Corresponding Author: Dr. Waheeta Hopper^1^*

Department of Bioinformatics,

School of Bioengineering,

Faculty of Engineering & Technology,

SRM University,

Kattankulathur - 603203,

Tamilnadu, India

E-mail: [hod.bioinfo@ktr.srmuniv.ac.in](mailto:hod.bioinfo@ktr.srmuniv.ac.in); [srmbioinforesearch@gmail.com](mailto:srmbioinforesearch@gmail.com)

Phone: +91-44-27417813

Fax: +91-44-27452343

**Supplementary Table 1**. Common pharmacophore hypotheses and model of the known efflux substrates of MexB and AcrB efflux systems

| **Hypotheses** | **Pharmacophore Model** | **Survival score** | **No of phytochemicals screened** |
| --- | --- | --- | --- |
| AADHR | 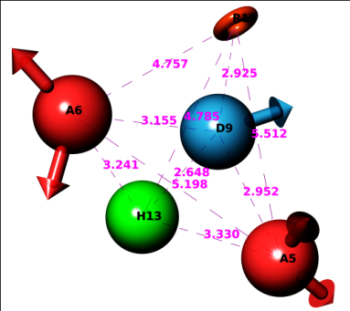 | 3.53 | 10 |
| ADHNR | 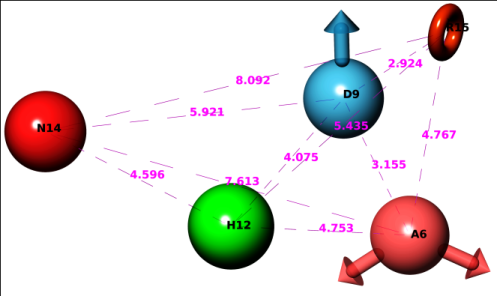 | 3.46 | 9 |
| AAHNR | 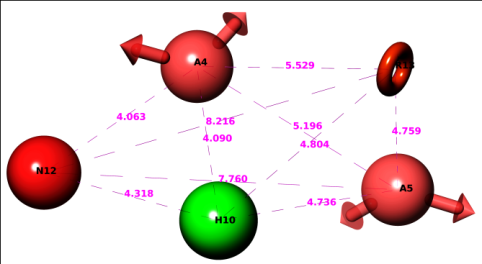 | 3.45 | 4 |
| AADHN | 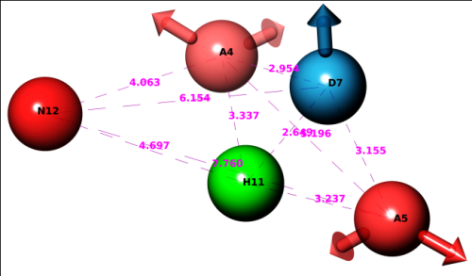 | 3.44 | 1 |
| AADNR | 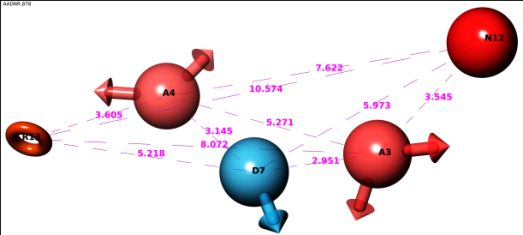 | 3.43 | 4 |
| AAADN | 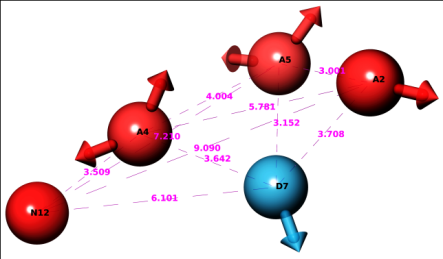 | 3.42 | 11 |
| AAADR | 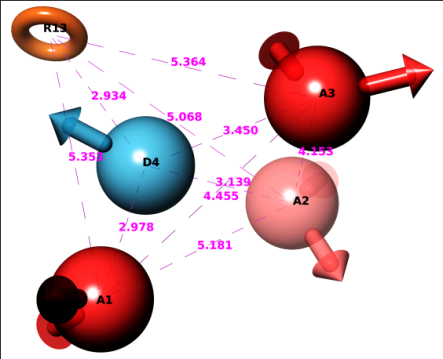 | 3.35 | 23 |
| AAANR | 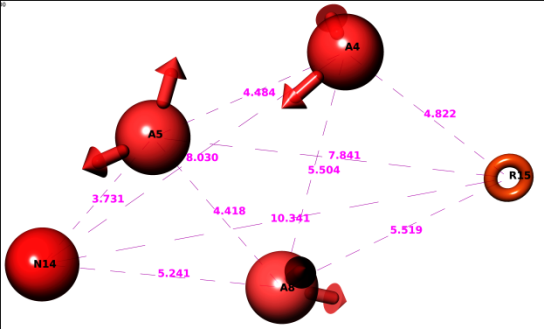 | 3.25 | 10 |
| AAAHN | 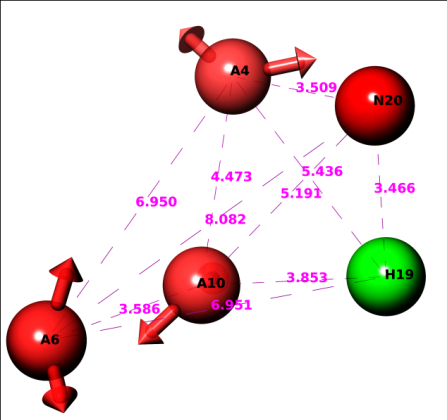 | 3.17 | 33 |
| AAADD | 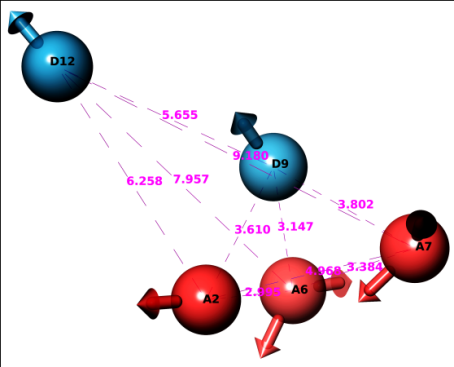 | 3.08 | 5 |
| AAADH | 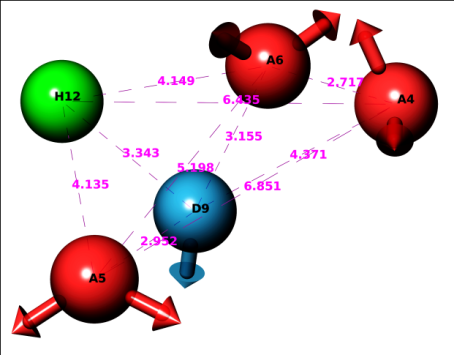 | 2.87 | 44 |
